# Button Cystostomy in Children with Neurogenic Bladder: Outcomes from a Single Center

**DOI:** 10.3390/jcm14155532

**Published:** 2025-08-06

**Authors:** Michela Galati, Rebecca Pulvirenti, Ida Barretta, Noemi Deanesi, Chiara Pellegrino, Antonio Maria Zaccara, Maria Luisa Capitanucci, Giovanni Mosiello

**Affiliations:** 1 Division of Neuro-Urology, Bambino Gesù Children’s Hospital, IRCCS, ERN eUROGEN Affiliated Center, Piazza di Sant’Onofrio 4, 00165 Rome, Italygiovanni.mosiello@opbg.net (G.M.); 2 Department of Neurosciences, Rehabilitation, Ophthalmology, Genetics, Maternal and Child Health (DiNOGMI), School of Medicine, University of Genoa, Largo Paolo Daneo 3, 16132 Genoa, Italy; 3 Department of Urology, Campus Bio-Medico University of Rome, via Alvaro del Portillo 21, 00128 Rome, Italy

**Keywords:** neurogenic bladder, urinary derivation, button cystostomy, endoscopy, bladder management, urinary tract infection

## Abstract

**Background**: Neurogenic bladder (NB) in children may lead to recurrent urinary tract infections (UTIs), renal deterioration, and a reduced quality of life. Clean intermittent catheterization (CIC) is the standard of care, but in some patients, CIC may be unfeasible due to anatomical, sensory, or compliance issues. Button cystostomy (BC) has emerged as a minimally invasive, bladder-preserving alternative. This study aimed to assess the feasibility, safety, and outcomes in the long-term of BC in pediatric NB patients. **Methods**: Retrospective analysis was conducted on children with NB who underwent endoscopic BC placement between January 2020 and December 2024 in a tertiary pediatric center. Demographic data, operative time, complications, and follow-up outcomes were collected. All procedures used an endoscopic approach with cystoscopic guidance for safe device placement. **Results**: Thirty-three patients (25 males; median age 7.96 years) underwent BC placement. Most had spinal dysraphism (63.6%). The mean operative time was 48.5 ± 6 min. During a mean follow-up of 2.1 ± 1.4 years, five patients (15.2%) had febrile UTIs and two had minor leakage. No major complications occurred. Four buttons were removed due to clinical improvement (N = 1), the fashioning of a continent derivation (N = 1) and implantation of a sacral neuromodulator (N = 2); two patients accepted CIC. Satisfaction was reported by 93.9% of families. **Conclusions**: BC is an effective, minimally invasive alternative for urinary drainage in children with NB, even when compared to continent diversion techniques such as the Mitrofanoff, due to its lower invasiveness, greater feasibility, and lower complication rate. Broader adoption may be warranted, but prospective studies are needed to confirm long-term outcomes.

## 1. Introduction

Neurogenic bladder (NB) is a complex condition that may be related to congenital or acquired conditions such as spina bifida and other spinal dysraphism, spinal cord injury, cerebral palsy, and can result in significant long-term morbidity, including upper urinary tract deterioration, incontinence, and impaired quality of life [1,2]. In children with NB, the primary therapeutic goals are to maintain a low-pressure bladder, ensure complete urinary drainage, and prevent urinary tract infections (UTIs) [3,4].

Early and effective bladder management is therefore essential to preserve renal function and optimize functional outcomes. The first-line treatment for these patients is clean intermittent catheterization (CIC) with anticholinergics, which has been shown to maintain low bladder pressures and reduce the risk of UTI [5,6]. However, in some patients, CIC is not feasible due to anatomical reasons, such as urethral stenosis; functional reasons, such as increased urethral sensitivity; or psychosocial limitations [7]. In these cases, a urinary diversion is required.

Urinary diversion techniques can be broadly categorized into continent and non-continent options. Continent diversions include procedures such as the Mitrofanoff [8] and Monti [9] techniques, while non-continent methods include mainly vesicostomy. The choice depends on the patient’s clinical condition, underlying pathology, and level of mobility.

Preliminary studies suggest that button cystostomy (BC) is an effective method of bladder drainage in pediatric patients [10,11,12,13]. Compared to a suprapubic catheter, it appears more stable and therefore usable for longer periods; relative to indwelling catheters, it is associated with fewer UTIs; and in contrast to vesicostomy, it allows for bladder function preservation [14]. Additionally, the BC can be placed endoscopically, enabling shorter operative and anesthesia times.

Despite these advantages, published data on the use of BC remain limited, particularly in terms of standardized indications, long-term outcomes, and patient/caregiver satisfaction. The aim of this study is to report our experience with BC in managing NB dysfunction in children and to evaluate its efficacy as an alternative to other established bladder drainage modalities.

## 2. Materials and Methods

### 2.1. Patients

A retrospective analysis was conducted of all children with NB who underwent BC placement at our tertiary referral center between January 2020 and December 2024. Patients were included regardless of age, underlying diagnosis, or comorbidities, provided they had a confirmed diagnosis of NB and a failed CIC trial. Baseline demographic data, operative details, and short- and medium-term postoperative outcomes were collected from medical records.

### 2.2. Ethical Considerations

This was an observational retrospective study based on standard clinical practice. All procedures performed, including follow-up evaluations, were part of routine care, and no interventions outside standard protocols were undertaken. Prior to data collection, all participants or their legal guardians provided written informed consent for the anonymous use of clinical data for research purposes. The local ethics committee was notified of the study on 2 May 2025, and approved its conduct (RAP-2005-0006). The study was conducted in accordance with the principles of the Declaration of Helsinki.

### 2.3. Study Outcomes

The primary aim of this study was to assess the feasibility, safety, and tolerance of BC placement in a pediatric NB population. Secondary objectives included evaluating the effectiveness of BC as a bladder management strategy and analyzing its associated short- and medium-term outcomes, such as urinary tract infections (UTIs), leakage, and patient satisfaction.

### 2.4. Study Design

This was a single-center, retrospective study. Data were extracted from electronic and paper-based patient records, including operative notes and outpatient follow-up documentation. Due to the heterogeneity of the cohort, the study focused on descriptive analysis rather than formal hypothesis testing.

### 2.5. Surgical Procedure

**Figure 1:**
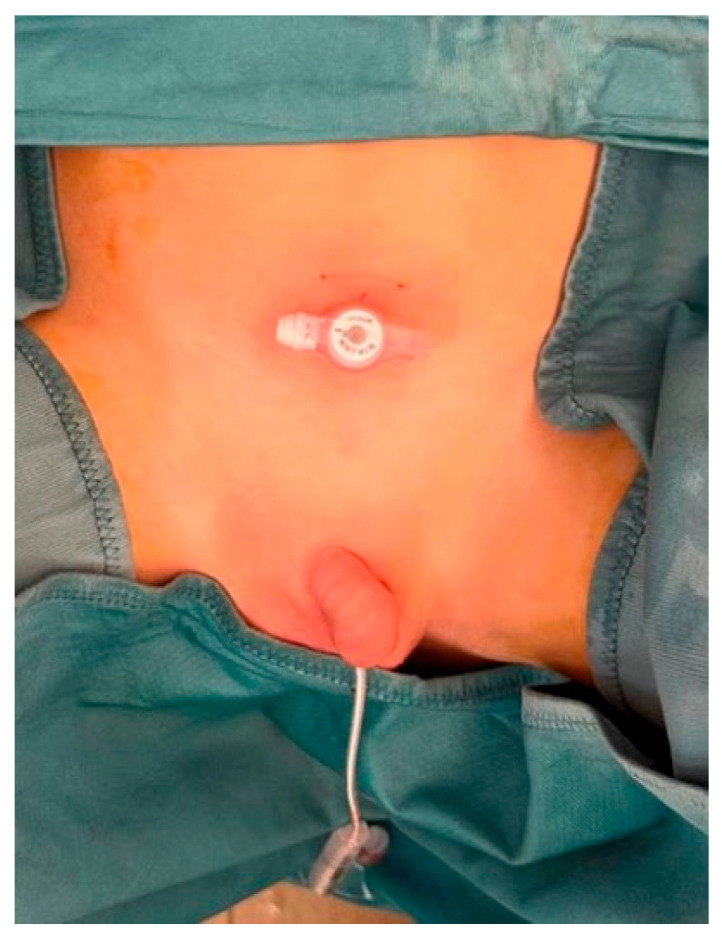
Final appearance of an endoscopically placed button cystostomy.

BC placement was carried out endoscopically under general anesthesia, following our modified Subramaniam’s technique [11,15,16]. After inserting a rigid cystoscope, the bladder was filled with isotonic saline. The bladder wall was fixed to the abdominal wall using an anchoring prolene suture. A 10 ch Cystofix^®^ (B. Braun, Bethlehem, PA, USA) needle was then introduced percutaneously in the suprapubic region to advance a guidewire into the bladder. The guidewire was retrieved through the urethra, the suprapubic tract was sequentially dilated, and an appropriate-sized cystostomy button was advanced and secured (Figure 1). In six patients who already had a suprapubic catheter in place, the existing tract was used to introduce the guidewire, facilitating the same endoscopic technique.

### 2.6. Follow-Up

Postoperative follow-up consisted of standard outpatient visits at 1, 3, 6, and 12 months. At each follow-up, standardized assessments were conducted to evaluate device tolerance, complications, exit-site leakage, and urinary tract infections (UTIs). Given the heterogeneity of the patient cohort (see Table 1)—characterized by stable yet severely impairing conditions—postoperative instrumental evaluations were tailored to individual clinical status. Specifically, invasive or non-invasive urodynamic studies were performed in patients for whom bladder function and pressure monitoring were clinically relevant. In contrast, for more compromised patients, an abdominal ultrasound or basic clinical evaluations, including serum creatinine testing, were preferred. Consequently, instrumental postoperative assessments were not considered as formal outcome measures in this study. Additionally, substitution of the first button was performed under anesthesia, while subsequent substitutions were conducted in the outpatient setting.

### 2.7. Statistical Analysis

Continuous variables are presented as median (interquartile range, IQR) or mean (standard deviation, SD), and categorical variables as counts and percentages. Statistical analyses were descriptive and conducted using SPSS version 25.0 (SPSS Inc., Chicago, IL, USA).

## 3. Results

A total of 33 patients with NB underwent endoscopic BC placement during the study period. The majority were males (25 males, 8 females).

The underlying diagnosis was primarily spinal dysraphism (SD) (N = 15). Other etiologies included NB secondary to spinal muscular atrophy type 1 (N = 1), surgical resection of bladder rhabdomyosarcoma (N = 1), polytrauma (N = 1), Currarino syndrome (N = 1), Bardet–Biedl syndrome with associated anorectal malformation and SD (N = 1), craniopagus twin (N = 1), SD associated with anorectal malformation (N = 2), VATER/VACTERL association including SD (N = 3), other neurologic diseases (N = 5), and other genetic syndromes (N = 5).

Bladder function was evaluated using an invasive and non-invasive urodynamic test in all patients, according to mental status and clinical conditions. Patients were primarily introduced to CIC, and the indication for endoscopic BC placement followed a failed CIC trial in all cases.

The median age at the time of BC placement was 7.96 years (IQR 5.22–13.96), and the median weight was 25 kg (IQR 16–35).

All procedures were performed endoscopically with no need for open conversion. The mean operative time was 48.54 +/− 6 min.

Following BC placement, all but one patient were enrolled in our standardized follow-up program.

The mean follow-up duration was 2.11 +/− 1.44 years. During this period, five patients (15.2%) experienced UTIs requiring antibiotic treatment, while two patients (6.1%) developed urinary leakage at the BC exit site, which was resolved by adjusting the button length and caliber. Further information can be found in Table 1.

Of the five patients who experienced UTIs, two had recurrent infections even prior to BC placement, while one patient developed a UTI only during an intensive care admission for aspiration pneumonia and permanent transurethral catheter placement.

A total of 31 out of 33 patients (93.9%) reported satisfaction with at-home BC management compared to CIC. The remaining two patients progressively accepted to perform CIC.

Four patients eventually had their BC removed after a mean duration of 2.6 +/− 1.28 years. In one case, this was due to conversion to permanent urinary diversion via a Mitrofanoff channel. One patient experienced clinical and instrumental symptom regression, allowing for BC removal. The remaining two patients underwent sacral neuromodulator implantation, which led to significant symptom improvement and enabled BC removal.

In one additional patient, BC removal was attempted following a favorable urodynamic study; however, the patient developed urinary retention, requiring button reinsertion.

## 4. Discussion

Our series reports on the use of BC for the management of NB patients, a technique that is being increasingly adopted at our institution due to encouraging clinical outcomes and positive feedback from both patients and caregivers. The ability to perform the procedure through a minimally invasive endoscopic approach highlights its advantages over more invasive urinary derivation techniques, further emphasized by the absence of conversions to open surgery in our series.

Based on our findings, BC allows for effective bladder management with a low rate of UTIs. In a systematic review by Jiang et al., the overall UTI rate in patients with NB was reported at 75.8%, decreasing to 24.2% when considering only recurrent infections [17]. Although preoperative UTI rates were partially unavailable in our cohort—due to the decentralized management of acute infections -, the postoperative UTI rate was 15.2% (or 12.1% if excluding the patient who developed a UTI during intensive care admission), which is notably lower compared to that reported in the literature.

This suggests that BC contributes to improved bladder control and may help reduce antibiotic use over time.

Historically, an alternative to BC in patients who either cannot tolerate or fail CIC has been suprapubic vesicostomy [18]. However, the vesicostomy procedure is more invasive, involves longer operative times and higher anesthesia risks, and results in a continuous urinary leakage with no potential to maintain bladder function—factors that can significantly impact patients’ daily activities and quality of life [19].

Supporting this, Nast et al. reported a lower rate of leakage following endoscopic BC placement compared to the open technique—a finding corroborated by our results, with a leakage rate of 6.1% versus the 71–100% reported by the authors in open procedures [19].

Moreover, BC placement is a quick procedure that results in minimal scarring and allows for the preservation of bladder capacity [15]. This is achieved by adjusting the duration the button remains closed, thereby promoting bladder filling. A key advantage of the suprapubic button is its versatility: it can be easily opened at regular intervals to permit spontaneous micturition, which helps maintain bladder tone and function.

BC can be used as either a permanent or temporary solution—e.g., as a bridge to bladder function recovery or while awaiting continent urinary derivation. It allows for standardized outpatient and home management, and its removal involves a minor surgical procedure [20]. Button replacements can be performed easily in an outpatient setting, avoiding the need for repeated anesthesia and hospital admissions.

Another advantage of BC placement is the absence of strict age restrictions for the procedure. Since cystoscopy is routinely performed across all pediatric age groups, BC can be safely placed even in very young patients. In our cohort, the youngest patient was 1.5 months old and weighed 6 kg. Similarly, the literature supports the use of BC across a broad age and weight range, without evidence of increased risks in infants [21].

However, as Lacreuse et al. noted, BC placement in small children with reduced bladder capacity and high intravesical pressure may be associated with increased complication rates, calling for careful patient selection [3].

Beyond the age criterion, BC also offers a practical and low-risk solution for neurologically impaired patients or those with significant surgical or anesthetic risks who may not be eligible for continent urinary diversion. The procedure avoids intestinal manipulation, thereby reducing the risk of adhesions and future bowel obstruction, while maintaining a relatively low rate of urinary tract infections.

To further support the broad applicability of BC, Table 2 summarizes the baseline characteristics and postoperative outcomes in our cohort compared to previously published case series. Notably, our data suggest a lower postoperative complication rate when BC is placed endoscopically, regardless of the patient’s age at the time of the procedure.

Finally, our findings, in line with the literature [22], highlight overall satisfaction with BC placement and usage. Although data using validated quality of life questionnaires remain limited, reported experiences suggest that BC improves patients’ daily life and activities, allowing participation in water sports and the usage of regular underwear instead of diapers [18].

A current limitation in BC use is the available device size, as the smallest commercially available button has a diameter of 12 Fr and the longest button measures 5 cm. This limitation primarily stems from the lack of a dedicated urinary-specific device, leading to the off-label use of gastrostomy buttons for this purpose [19,23]. Similarly, using a device designed for gastrointestinal applications presents challenges in the supply of compatible accessories and replacements.

In Italy, for example, the off-label nature of the device precludes its inclusion among home supplies reimbursed by the national healthcare system. As a result, only hospitals are authorized to provide the device, and patients must attend regular outpatient visits for button replacement. This contrasts with its approved use in gastrointestinal indications, where the device is routinely covered by the healthcare system and can be replaced at home by a dedicated nurse.

To support broader adoption, Makker et al. evaluated the annual maintenance costs of a mini-button gastrostomy per patient compared to clean intermittent catheterization (CIC), showing that buttons represent a lower financial burden [24].

While the present study specifically focuses on children with neurogenic bladder, our clinical experience with BC placement is expanding to include other patient populations, such as those with posterior urethral valves, where we are observing similarly favorable outcomes. These preliminary findings support the notion that BC may be effectively and safely utilized across a broader spectrum of urological conditions, potentially offering the same functional advantages and quality-of-life improvements beyond neurogenic indications.

Although our findings are encouraging, the use of a device not originally intended for urinary applications may pose risks, such as reduced durability, leakage, or device blockage, due to differences in material compatibility and exposure to urine. These potential issues, while not prominent in our series, warrant further investigation and underline the importance of regulatory scrutiny in off-label applications.

Furthermore, the limited size range of available gastrostomy buttons may hinder the transition to adult care for pediatric patients managed with the device. Despite emerging evidence supporting BC use in adults, the maximum available button length remains a limiting factor in older or larger patients [25].

Given the growing adoption of BC among pediatric urology centers and the promising clinical outcomes reported thus far, there is a clear need to develop a device specifically designed for urinary diversion. Such a device should be tailored to the anatomical and functional characteristics of the urinary tract. Further research demonstrating the efficacy, safety, and user satisfaction associated with BC could help drive the development of a dedicated product for both pediatric and adult patients. Future prospective and multicenter studies, incorporating long-term follow-up and validated quality-of-life assessments, are essential to confirm our findings and to establish evidence-based guidelines for the use of BC in pediatric neurogenic bladder.

### Strengths and Limitations

The main limitations of this study are its retrospective design and the relatively small sample size. In addition, the single-center nature of the study and the lack of a comparative group represent further methodological limitations. Furthermore, the absence of standardized quality-of-life questionnaires limits the ability to quantitatively assess patient and caregiver satisfaction—an area that remains underexplored. Lastly, the relatively short follow-up period constrains the strength of conclusions regarding long-term efficacy and durability.

Nonetheless, the study has several strengths, including a homogeneous cohort consisting exclusively of NB patients and a wide age range, both of which support the feasibility of BC placement and its potential for widespread clinical application in this patient population.

## 5. Conclusions

The use of BC in patients with NB appears to be a reasonable and effective option for urinary diversion. Our results, drawn from a homogeneous cohort across a wide age range, support the feasibility of the endoscopic approach and indicate a relatively low rate of postoperative complications. However, long-term outcome studies are needed to confirm the stability of BC as a method for bladder management. Comparative studies between BC and other incontinent diversion techniques may further clarify its potential role in preserving bladder function.

## Figures and Tables

**Table 1:** Baseline characteristics and postoperative outcomes for included patients.

Patient	Sex	Underlying Condition	Age at BC Placement (Years)	Weight at BC Placement	Postoperative Complications
1	M	VACTERL association	7.9	22	-
2	F	Chromosome 8 microdeletion	2.8	10	-
3	M	SD	19.4	75	-
4	M	ACTG2 gene mutation	6.5	19	leakage
5	F	Craniopagus twin	4.5	19	UTI
6	M	Encephalopathy	11.8	39	UTI
7	M	VACTERL association	14.5	40	-
8	M	Nicolaides–Baraitser syndrome	5.4	21	leakage
9	M	SD	0.15	6	-
10	M	Epileptic encephalopathy	15.8	16	UTI
11	M	SD	14.8	42	-
12	M	SD associated with ARM	4.7	16	-
13	M	SD	7.3	35	-
14	M	Currarino syndrome	15.5	30	-
15	F	VACTERL association	7.4	16	-
16	F	Complex brain malformation	8.6	25	-
17	M	Chromosomal rearrangement	15.9	48	-
18	M	Acute encephalomyelitis	12.9	30	UTI
19	M	SD	5.5	13	-
20	M	Spinal muscular atrophy	10.2	26	-
21	M	Bardet–Biedl syndrome	2.9	16	UTI
22	M	Bladder rhabdomyosarcoma	9.6	27	-
23	F	SD	5.1	15	-
24	F	SD	7.7	13	-
25	F	Crouzon syndrome	8.9	25	-
26	M	SD associated with ARM	0.7	4	-
27	M	SD	14.8	53	-
28	M	SD	5.5	22	-
29	M	SD	7.9	16	-
30	F	SD	4.2	35	-
31	M	Polytrauma	16.3	45	-
32	M	Cerebral ischemia	13.2	30	-
33	M	SD	13.5	35	-

M = male; F = female; SD = spinal dysraphism; ARM = anorectal malformation; UTI = urinary tract infection.

**Table 2:** Comparison of patient characteristics, surgical techniques, and postoperative outcomes in our study cohort versus published series.

	Study Cohort (N = 33)	Lacreuse et al. [3] (N = 21)	Varik et al. [18] (N = 29)	Nast et al. [19] (N = 13)
Age at BC placement (y)	9.14	6.8	5.04	5
BC placement technique	EBC: 33 (100)	EBC: 21 (100)	OBC: 14 (48.3) EBC: 3 (10.3) EV: 5 (17.2) SPC: 7 (24.2)	OBC: 1 (7.7) EBC: 5 (38.5) EV: 7 (53.8)
Postoperative complications	2 (6.1)	7 (33.3)	10 (34.4)	9 (69.2) *
Postoperative UTI	5 (15.2)	4 (19)	9 (31)	3 (23)

EBC = Endoscopic button cystostomy placement; OBC = Open button cystostomy placement; SPC = Previous supra-pubic catheter; EV = Existing vesicostomy. * Stoma leakage was reported in all patients undergoing open BC placement or with a pre-existing vesicostomy; no leakage was reported in patients undergoing endoscopic BC placement.

## Data Availability

The original contributions presented in this study are included in the article. Further inquiries can be directed to the corresponding author(s).

## Abbreviations

The following abbreviations are used in this manuscript:

NB	Neurogenic bladder
UTI	Urinary tract infection
CIC	Clean intermittent catheterization
BC	Button cystostomy
SD	Spinal dysraphism
IQR	Interquartile range
